# Predictors of Citation Rate in Psychology: Inconclusive Influence of Effect and Sample Size

**DOI:** 10.3389/fpsyg.2017.01160

**Published:** 2017-07-11

**Authors:** Paul H. P. Hanel, Jennifer Haase

**Affiliations:** ^1^School of Psychology, Cardiff University Cardiff, United Kingdom; ^2^Department of Psychology, University of Bath Claverton Down, United Kingdom; ^3^Department of Psychology, Lund University Lund, Sweden

**Keywords:** citation rate, citation frequency, effect size, sample size, power, open access, journal impact factor, statistical significance

## Abstract

In the present article, we investigate predictors of how often a scientific article is cited. Specifically, we focus on the influence of two often neglected predictors of citation rate: effect size and sample size, using samples from two psychological topical areas. Both can be considered as indicators of the importance of an article and *post hoc* (or observed) statistical power, and should, especially in applied fields, predict citation rates. In Study 1, effect size did not have an influence on citation rates across a topical area, both with and without controlling for numerous variables that have been previously linked to citation rates. In contrast, sample size predicted citation rates, but only while controlling for other variables. In Study 2, sample and partly effect sizes predicted citation rates, indicating that the relations vary even between scientific topical areas. Statistically significant results had more citations in Study 2 but not in Study 1. The results indicate that the importance (or power) of scientific findings may not be as strongly related to citation rate as is generally assumed.

## Introduction

Citation rates (CRs) often serve as a quantitative indicator of a researcher’s performance. They are employed heuristically to evaluate the utility of publications, the importance of the journals that publish them (through Journal Impact Factor, JIF), and the scientific success of the authors who write them (Leimu and Koricheva, 2005). Thus, it is of great interest to better understand what CRs consist of, that is, whether they are predicted only by the novelty, quality, importance, and significance of a paper or if other, less substantial, factors have an influence, too.

Ideally, a paper should be cited when it is of high quality, importance, and contains useful and novel information. In practice, however, many other factors also influence the CRs of articles. Because the reading-time of researchers is limited (Niu and Hemminger, 2012), while the number of new articles is rapidly increasing, scientists have to decide what to read and, thereafter, what to cite in their own publications. This makes it more likely that researchers rely, at least to some degree, on shallow attributes of the articles when making these decisions (Petty and Cacioppo, 1986). Indeed, research shows that CRs provide only an “illusion of objectivity” (Leimu and Koricheva, 2005, p. 28) because they can be predicted by numerous subjective and social factors that are unrelated to the quality and importance, too (for a list of predictors, see **Table 1**). There are several predictors of CR whose predictive effect could be replicated, such as free access to the paper or the number of references (but see Gaulé and Maystre, 2011 for the former). However, there are also several factors for which predictive findings are mixed, such as the number of pages, the rank of the main authors’ institutes or their reputation, as measured by the total number of citations (cf. **Table 1**).

**Table 1:** Overview of several predictors of citation rate.

Factor	Positive relation to citation frequency	No relation to citation frequency
Impact factor	Callaham et al., 2002; Leimu and Koricheva, 2005; Judge et al., 2007; Etter and Stapleton, 2009; Mingers and Xu, 2010; Hegarty and Walton, 2012; Didegah and Thelwall, 2013a,b; Uthman et al., 2013; Vanclay, 2013	/
Number of references	Peters and van Raan, 1994; Judge et al., 2007; Kostoff, 2007; Mingers and Xu, 2010; Didegah and Thelwall, 2013a; Farshad et al., 2013; Hanssen and Jørgensen, 2014	/
Impact of articles which are cited in the paper	Baldi, 1998; Didegah and Thelwall, 2013a,b	/
Paper awarded	Stremersch et al., 2007	/
Industrial support	Kulkarni et al., 2007; Etter and Stapleton, 2009; Farshad et al., 2013	/
Self-citation rates	Stremersch et al., 2007	/
Publication record	Stremersch et al., 2007	/
Citation record	Leimu and Koricheva, 2005; Bergh et al., 2006	/
Editorial Board Membership	Stremersch et al., 2007	/
Report positive results	Fanelli, 2013	/
Open access	Lawrence, 2001; Piwowar et al., 2007; Vanclay, 2013	After controlling for quality: Gaulé and Maystre, 2011
Position in Journal	Laband and Piette, 1994; Ayres and Vars, 2000; Leimu and Koricheva, 2005; Stremersch et al., 2007	/
Number of authors	Baldi, 1998; if *N* > 4; Peters and van Raan, 1994; Leimu and Koricheva, 2005; Bergh et al., 2006; Kostoff, 2007; Hegarty and Walton, 2012; Didegah and Thelwall, 2013b; Uthman et al., 2013; Vanclay, 2013; Hanssen and Jørgensen, 2014; Wang and Shapira, 2015	Didegah and Thelwall, 2013a
Title length	For shorter titles: Ayres and Vars, 2000; Hanssen and Jørgensen, 2014; for longer titles: Farshad et al., 2013	Stremersch et al., 2007; Haslam et al., 2008; Didegah and Thelwall, 2013a
Number of countries involved	Mingers and Xu, 2010; Hanssen and Jørgensen, 2014	Didegah and Thelwall, 2013a
Position first letter from first Author in Alphabet	Tregenza, 1997	Leimu and Koricheva, 2005; Hegarty and Walton, 2012; Vanclay, 2013; Hanssen and Jørgensen, 2014
Gender	With more cites for female authors: Ayres and Vars, 2000; with less cites for female authors: Baldi, 1998	Leimu and Koricheva, 2005; Judge et al., 2007; Hanssen and Jørgensen, 2014
Graph density	/	Negative correlations: Hegarty and Walton, 2012
Table density	Hegarty and Walton, 2012	/
Model density	Hegarty and Walton, 2012	/
Sample size	Callaham et al., 2002; Kostoff, 2007; Farshad et al., 2013	Leimu and Koricheva, 2005; Bergh et al., 2006; Etter and Stapleton, 2009; Barto and Rillig, 2012; Hegarty and Walton, 2012
Effect size	Leimu and Koricheva, 2005; Barto and Rillig, 2012	/
Significant results cited more often	Etter and Stapleton, 2009	/
Sharing detailed research data	Piwowar et al., 2007	/
Rank of Institute	Leimu and Koricheva, 2005; Bergh et al., 2006; Stremersch et al., 2007; Mingers and Xu, 2010	Haslam et al., 2008
Reputation of first Author	Peters and van Raan, 1994; Leimu and Koricheva, 2005; Haslam et al., 2008; Vanclay, 2013	Baldi, 1998; Judge et al., 2007; Farshad et al., 2013
Number of pages	Bergh et al., 2006; Kostoff, 2007; Mingers and Xu, 2010; Hegarty and Walton, 2012; Farshad et al., 2013; Vanclay, 2013; until *N* < 54: Ayres and Vars, 2000	Leimu and Koricheva, 2005; Judge et al., 2007
Reliability demonstrated	Judge et al., 2007	
General quality judgment by experts	Gottfredson, 1978; Eyre-Walker and Stoletzki, 2013	
Novel idea within (sub-) field	Judge et al., 2007	Newman and Cooper, 1993


As can be seen in **Table 1**, the majority of the investigated predictors are not directly related to the novelty, quality, or importance of a paper. Examples include number of pages, references, or authors, and title length. We call those less substantial predictors because they are only indirectly related to or unrelated to the quality, novelty, and importance of a paper.

In this article, we focus on two often neglected predictors of CR: effect and sample size. Both should predict CRs well, as we outline below. Furthermore, we consider it as essential to establish the combined influence of more and less substantial predictors to assess their relative and independent contribution to CRs.

Why should effect and sample sizes predict CR? We argue that both, larger effect and larger sample sizes, are – independently as well as interactively – indicators of greater importance of any study, at least in specific topical areas (see below). Assume for example that researcher 1 develops a method to reduce prejudices (an outcome that is usually considered as beneficial). The result is statistically significant with an effect size of d1 (e.g., between experimental and control group). Researcher 2 develops at the same time independently an improved method with the effect d2, whereas d2 = 3^∗^d1 (assuming equal sample sizes across both studies). Consequently, the paper of researcher 2 should be cited more often, for example by other researchers who also seek to employ methods to reduce prejudices. Furthermore, most scientific psychologists seem to be interested in positive findings, i.e., those which confirm their hypothesis (Nosek et al., 2012). Indeed, over 90 percent of the papers published in psychology/psychiatry report data in favor of the hypothesis – more than in any other scientific field (Fanelli, 2010). Large effect sizes are more likely to be statistically significant. Hence, researchers should have an interest in selecting those methods which result in larger effect sizes for their own research, as this will more likely result in a significant outcome and therefore publication (e.g., when testing the effects of specific emotion regulation strategies in another sample or probing a method to reduce prejudice in another context). Nevertheless, there may be several exceptions where a smaller effect-size may be more desirable, at least to some researchers. We address this issue below.

A larger sample size should predict CR because it is associated with a larger *post hoc* (or observed) power, making the findings more convincing. Although one study about the predictors of CR defined quality as sample size (Barto and Rillig, 2012), this definition of quality neglects several other facets of quality (e.g., rigorousness of the design, procedure, or data analysis). Instead, effect and sample size seem to us indicators of the *importance* of a study and simultaneously of power. A large effect size is likely to be an indicator of an accurate method, for example because of low error variance, and/or a different approach toward a specific problem, compared to small effect sizes within the same topical area. A large sample size increases the statistical power, always *ceteris paribus* (i.e., with all other variables held constant).

The focus on effect and sample size is also relevant in light of the current replication crisis within psychology, as underpowered studies lead, especially in combination with publication bias, to problematic outcomes such as an overestimation of effect sizes and low reproducibility of findings (Button et al., 2013; Open Science Collaboration, 2015). If sample and effect size correlate with CR, this would indicate that although power is often neglected while designing a study (Sedlmeier and Gigerenzer, 1989; Cohen, 1992), it is considered and acknowledged through citation.

There are several other profound and substantial factors that should theoretically predict CRs as well, beside effect and sample size. Examples include the quality of a study as operationalized by the apparatus and material used, procedure, demonstrated reliability or robustness of the statistical analysis, and discussions of limitations and implications. However, given only weak predictions and inconclusive findings of those quality variables (Newman and Cooper, 1993; Judge et al., 2007), measuring them seems in general difficult. This view is supported by low inter-rater reliabilities of quality assessments – even among experts in the same topical area (Bornmann et al., 2010; Eyre-Walker and Stoletzki, 2013). If researchers cannot agree on quality – at least not in a way that can be simplified to statistical markers – it is unlikely that quality measures will predict CR. In contrast, effect and sample size can be quantified in a simple way and a rationale given for why they should be linked to CR (see above).

Only two studies have investigated the relation between effect sizes and CR so far (Leimu and Koricheva, 2005; Barto and Rillig, 2012), to the best of our knowledge. Both sampled publications in the field of ecology and have found small positive correlations, on average, depending on the topical area and the hypothesized direction of effect. In contrast, sample size was a positive predictor of CR, but only in some scientific (sub-)fields (Callaham et al., 2002; Leimu and Koricheva, 2005; Bergh et al., 2006; Kostoff, 2007; Etter and Stapleton, 2009; Barto and Rillig, 2012; Farshad et al., 2013).

However, most of these studies did not report the unique influence of the effect and sample size, i.e., controlled for other factors which can have an influence on the sample and effect size, as well as the CR, such as the JIF. Furthermore, whether smaller effect sizes are desirable in specific topical areas or cases was not controlled, adding noise to the data. In the present research, we aim to overcome those limitations. In the past, it was shown that different scientific fields have different publications and citation traditions (Didegah and Thelwall, 2013b; Fanelli, 2013), therefore it seems relevant to investigate the afore mentioned hypotheses within a different field, such as psychology.

In psychology we identified two studies that have investigated numerous predictors of CRs (Haslam et al., 2008; Hegarty and Walton, 2012). In the first study, Haslam et al. (2008) considered a wide range of variables (such as the JIF, title length, or design). They did not report any findings on variables that are more closely related to the quality or importance of an article. Another difference to our approach is that we do not use journals (Haslam et al., 2008), but two specific topical areas within psychology to sample our data. This is because comparisons of effect sizes even across topical areas are often not meaningful, given that the interpretation of effect sizes can strongly rely on the context (Sharpe, 2013). In the second study which has investigated predictors of CR within psychology, Hegarty and Walton (2012) did not find a relation between the number of participants and the CR, but a somewhat surprisingly negative correlation of the sample size with the JIF.

Two further aims of this study were to partly replicate previous findings and to shed more light on some preceding inconclusive findings (cf. **Table 1**). We are especially interested in whether freely available articles as well as such that report statistically significant results are cited more often and whether the strong effect of the position of the first letter of the first author’s name in the alphabet can be replicated (Tregenza, 1997).

## Overview of the Present Studies

Our aim was to sample articles from topical areas of psychology where larger effect sizes can be considered as more beneficial. To get an exhaustive sample, we searched for large meta-analyses with tables that contained effect and sample size of each study. We included in our search Google Scholar (GS), recent issues of two journals which are known to publish meta-analyses (Psychological Bulletin along with Personality and Social Psychology Review), and meta-analyses we were familiar with. We used meta-analyses to select our studies because (a) the studies included have been considered as relevant by experts in a specific topical area and (b) the relevant statistics have been derived from all studies within each meta-analysis in the same or at least a very similar way. We stopped our search after two large meta-analyses were found for which we think that there are good reasons to argue that larger effect sizes are more beneficial. The first meta-analysis has analyzed 190 studies of emotion regulation strategies (ERS; Webb et al., 2012), the other 515 studies about whether and how intergroup contact reduces prejudices (Pettigrew and Tropp, 2006). In both cases, larger effect sizes indicate a stronger effect and therefore a better efficacy of a method. This can have immediate practical consequences and is therefore more relevant than studies reporting smaller effect sizes. Consequently, they should be cited more often. For example, effective ERS can be useful within clinical and organizational contexts. However, it is possible that this effect is stronger for those studies which reported effect sizes relevant to ERS (or reduction of prejudice), as researchers don’t need to rely on rule of thumbs to estimate effect sizes based on descriptive statistics (e.g., mean and standard deviation) and/or test statistics (e.g., *t*- and *p*-value along degrees of freedom).

To ensure that effect sizes selected were those related directly to the principle research questions of the articles, we selected in a second step only those papers in which the topics of the meta-analyses were mentioned in the title of each paper. This strategy had two underlying assumptions: that the main content of an article can be found in its title and that most researchers decide what to read based on the title. The reporting standards of the American Psychological Association, which are used by most psychological journals, clearly ask that the main topic under investigation is summarized in the title (American Psychology Association [APA], 2010). Given an average reading time of 11 h per week among academics (Niu and Hemminger, 2012) and a large volume of scientific publications it seems reasonable to assume that academics must employ heuristics in making initial selections, like using the title as a very first criterion for the subjective relevance of an article. Taking two articles from the meta-analysis about ERS (Webb et al., 2012) as an example, an article titled “Behavioral inhibition and amplification during emotional arousal: A comparison of two age groups” (Kunzmann et al., 2005) was excluded, because it would have been unclear whether citing articles would cite it because of differences between ERS or because of differences between the two age groups within one ERS. On the other hand, an article with the title “Impact of rumination versus distraction on anxiety and maladaptive self-beliefs in socially anxious individuals” was included, because the title made it clear that two ERS are compared and did not refer to any other major findings (Wong and Moulds, 2009).

Papers were only included when the reference could be found in the search engine GS^1^. The data files for both studies are stored on the open science framework^2^.

## Study 1

### Method

#### Materials

We included 140 studies (73.68%) from a meta-analysis about the effectiveness of ERS (Webb et al., 2012). Studies were selected when the title of the paper referred directly to emotion regulation strategies (see above for a rationale and an example). Ninety-two studies (65.7%) were freely available on GS, 103 (73.6%) were single-study articles, 80 (57.1%) reported only one focal test, and 57 (41.4%) papers included an effect size. Of those 57 papers, 24 reported Cohen’s *d*, 13 eta square, 14 partial eta square, 1 eta, and 5 standardized betas. Several papers included both (partial) eta square for a two- or three-way ANOVA and Cohen’s *d*s for the pairwise comparisons (e.g., control group vs. ERS group). Here we coded the effect sizes as Cohen’s *d*, because pairwise comparisons are more relevant to estimate the effectiveness of ERS and also easier to interpret than multiple group comparisons. All 140 studies were included in the principal analysis, but we repeated the analysis in several subsets of this sample (e.g., only single-study papers or studies that have reported effect sizes). A formal power-analysis was not conducted because our selection criteria described above restricted the overall sample size.

#### Variables

For each paper, the largest effect size, Cohen‘s *d*, and the associated number of participants, were selected from the meta-analysis (Webb et al., 2012). In most of the studies for which multiple outcomes were reported, several ERS were compared on a specific emotion (e.g., anxiety or sadness). We selected the largest effect size because we assumed that when other researchers are deciding which methods for ERS they would use in their own research, they would compare various studies and select the most efficient ERS (i.e., the one with the largest effect size). However, as this procedure can add some noise, we also analyzed the papers which reported only one study and one test relevant to ERS separately (more than one relevant test usually meant that several ERS’ were compared within a single-study). Both effect size and sample size were used to estimate the *p*-value for each sample, assuming equal sample sizes. This was done because barely any of the original studies reported an exact *p*-value. The number of authors, the journal, and the year of publication were collected from the references directly.

The 2-year impact factor for each journal was collected from Web of Science (WoS)^3^, or the website of the publisher, using data from 2015. The search engine GS was used to extract the number of citations (as of March 2017) and to check whether the papers were freely accessible on the internet. We did not distinguish whether the article was made legally available by the publisher or whether someone has made copyright material available online. GS can be considered as a valid source of CR because unlike other citation services, it not only takes the citations of specific journals into account but also book (chapter)s, conference proceedings, or non-English language journals. However, GS CR may be inflated (Meho and Yang, 2007). Therefore, we decided to use additionally the CR as provided by WoS as a dependent variable. We used WoS instead of Scopus, another widely used bibliographic database, because WoS was easier accessible for us. We do not expect that using Scopus would have brought different results, as the output of both databases is very similar (Archambault et al., 2009).

Because the distributions of most variables are skewed, we transformed all our variables using rank-based inverse normal transformation (except free access, because this variable is dichotomous). This transformation can normalize most distribution shapes through “converting the data into ranks, similar to the Spearman approach, but then converting the ranks into probabilities, and finally using the inverse cumulative normal function to convert these probabilities into an approximately normal shape” (Bishara and Hittner, 2012, p. 401). Further, it has been found to be more beneficial over 11 other methods to handle non-normality in terms of Type I and Type II error control with sample sizes ≥ 20 (Bishara and Hittner, 2012). A rank-based inverse normal transformation results in a monotonic (usually non-linear) relation between the raw and transformed variable with a mean of 0 and a standard deviation of 1. The transformations were done in R with the package GenABEL, version 1.8-0 (Aulchenko et al., 2007).

### Results

#### Correlational Analyses

First, the correlations of the eight predictors with the CRs as provided by GS and WoS as the dependent variable were computed, using the transformed variables. As can be seen in **Table 2**, neither the effect size nor sample size correlated statistically significantly with the CR. The pattern of results was the same when Spearman’s rank correlation coefficients was used with the untransformed variables. However, despite the overall effect size was positive, some studies reported a negative effect size (29 studies or 20.7%). That is, the control group scored higher than the experimental group. This might have affected the results. We therefore first used the absolute value of the effect sizes and, in a second step, excluded all studies with negative effect sizes from the analysis. However, effect and sample sizes remained uncorrelated with the two measures of CRs. Also, no effects were found when we only focused on the single-study papers, papers reporting only one effect relevant to ERS, or papers reporting effect sizes, neither when we computed Pearson’s correlations with the transformed variables nor Spearman’s with the untransformed variables. In a final step, we excluded the 13 studies reporting effect sizes > 1.50, as effect sizes of this magnitude can indicate poor quality, because they are very unlikely to occur, given the distribution of effect sizes in psychology (Richard et al., 2003; Gignac and Szodorai, 2016). However, the pattern of results remained unchanged.

**Table 2:** Correlation between each variable (Study 1).

		*M*	*SD*	1	2	3	4	5	6	7	8	9
1	Citation rate GS	252.37	390.19	1								
2	Citation rate WoS	114.20	165.16	0.99	1							
3	Effect size *r*	0.46	0.69	0.12	0.14	1						
4	*p*-value	0.12	0.15	-0.10	-0.11	-0.64	1					
5	Sample size	61.00	44.96	0.08	0.08	0	-0.22	1				
6	Free access	0.66	0.48	0.40	0.40	0.18	-0.1	-0.02	1			
7	Journal impact factor	3.21	1.26	0.46	0.50	0.08	-0.05	-0.07	0.23	1		
8	Number of authors	2.92	1.35	0.00	0.01	0.25	-0.09	-0.08	0.17	-0.01	1	
9	Position of 1st letter	12.09	6.82	-0.40	-0.41	0.10	-0.02	0.05	0.05	-0.15	0.23	1
10	Year of publication	2004.66	5.19	0.03	0.05	0.04	0.02	0.05	-0.03	-0.08	-0.19	0.17


*In order to avoid the focus on statistically significant results based on the NHST (e.g., Salsburg, 1985; Masicampo and Lalande, 2012), the usual significance asterisks has been omitted in the table. Every |*r*| > 0.165 is significant on *p* < 0.05, |*r*| > 0.217 on *p* < 0.01 and every |*r*| > 0.275 on *p* < 0.001, two-tailed. *N* = 140. All variables are RIN transformed except ‘Free access’ before correlations were computed. GS, Google Scholar; WoS, Web of Science; Free availability, 1: Articles is freely available, 0: is not; position of 1st letter: Position of the first letter of the surname of the first author in the alphabet.*

#### Multiple Regressions

A multiple regression with CRs as the dependent variable and the other seven variables as predictors, returned similar results as the correlational analyses (**Table 3**): The beta for effect size was 0.07 (*p* = 0.27) and for sample size 0.14 (*p* = 0.045). The overall adjusted *R*^2^ was 0.45 (*F*[7,131] = 17.06, *p* < 0.0001), with the year of publication (β = -0.42), free access on the internet (β = 0.68), and the journal impact factor (β = 0.35) as the other significant predictors (*p*s < 0.001). The results hold when CR from WoS were used and when only those studies reporting an effect size and single-study papers were included. However, contrary to our prediction, sample size did not reach statistical significance anymore when only those articles were included, which reported one focal test (β = 0.05, *p* = 0.56). In none of these additional analyses did effect sizes predict CRs. The results for CRs as provided by WoS were very similar. *P*-values were not included as predictors, because they are a composite score of effect and sample size, reducing their unique influence which we considered as more relevant. In a final step, we excluded again the 13 studies reporting effect sizes > 1.50. However, the pattern of results remained unchanged.

**Table 3:** Predictors of citation rate (multiple regression analyses).

	Study 1		Study 2	
		
Variable	*B*	95% CI	*B*	95% CI
Constant	-0.44	[-0.66, -0.22]	-0.07	[-0.25, 0.10]
Sample size	0.13	[0.00, 0.26]	0.30	[0.14, 0.46]
Effect size	0.05	[-0.08, 0.18]	-0.17	[-0.34, -0.00]
Journal Impact Factor	0.35	[0.22, 0.48]	0.45	[0.29, 0.60]
Number of authors	0.07	[-0.07, 0.22]	0.11	[-0.08, 0.30]
Year of publication	-0.42	[-0.55, -0.28]	0.22	[0.07, 0.38]
Position of 1st letter	0.14	[0.01, 0.28]	-0.10	[-0.26, 0.06]
Free access	0.68	[0.41, 0.96]	0.33	[-0.17, 0.82]
Adjusted *R^2^*	0.45		0.32	
*F*	17.06		9.33	


**N* = 161. All variables are RIN transformed except ‘Free access.’ Citation rate: As provided by Google Scholar, Free access: 1: Articles is freely available, 0: is not; Position of 1st letter: Position of the first letter of the surname of the first author in the alphabet. CI, Confidence Interval. Study 1: sample size and position of 1st letter are significant at *p* = 0.045 and *p* = 0.049, journal impact factor, year of publication, free access, and *F*-value are significant at *p* < 0.001. Study 2: sample size, impact factor, and *F*-value are significant at *p* < 0.001, year of publication at *p* = 0.006, effect size at *p* = 0.045.*

#### Interaction of Effect and Sample Size

Because it is possible that there is an interaction between effect and sample size, for example in the sense of that only studies that report larger effect *and* larger sample sizes are cited more often predict CR, we did regression analyses with an interaction term, using the non-transformed variables. None of the interaction terms was significant, neither for all articles combined, those reporting one study, one focal test, one effect sizes, and studies reporting positive effects (*ds* > 0), nor when the CRs as provided by WoS were used (all βs < 0.37, *p*s > 0.11).

#### Significant vs. Non-significant Results

In a next step, articles reporting significant versus non-significant results were compared using independent sample *t*-tests. *P*-values were estimated based on a directed hypothesis, i.e., we assumed that the null-hypothesis significance testing was done based on a one-tailed test. No statistical significant differences were found, neither for all articles combined, those reporting one study, one focal test, one effect sizes, and studies reporting positive effects (*ds* > 0), nor when the CRs as provided by WoS were used (all |*t*s| < 1.33, *p*s > 0.18). The results remained the same when *p*-values were estimated based on a two-tailed test.

#### Exploratory Analysis

Further exploratory analyses compared freely available articles with those that were not freely accessible, using all other transformed variables as dependent variables (see **Table 2**). A MANOVA was significant, *F*(9,127) = 4.75, *p* < 0.001, $\eta_{p}^{2}$ = 0.24. Follow-up between-subject *t*-tests revealed significant differences between three variables. Freely accessible articles were cited more often, had larger effect sizes, were published in journals with higher IFs, and had more authors (*ds*: 0.91, 0.39, 0.49, 0.36, all *p*s < 0.035). The differences of the other four variables were not significant (*p*s > 0.20; all tests two-tailed). The results hold for WoS CR.

## Study 2

The main aim of Study 2 is to replicate the findings of Study 1 in another psychological topical area, because it is important to reproduce also non-significant findings (risk of false-negative results).

### Method

#### Material

A meta-analysis of the contact hypothesis was chosen for sampling articles (Pettigrew and Tropp, 2006). The contact hypothesis states that intergroup contact reduces intergroup prejudice. Larger negative effect sizes in these studies evidence the efficacy of contact in reducing prejudice. Reduction of prejudice is generally considered a beneficial outcome. Therefore, being beneficial, such a study should be cited more often than less beneficial studies. In the meta-analysis, all effect-sizes have been transformed to correlations for the purpose of comparisons by Pettigrew and Tropp (2006). A negative correlation indicates a more favorable outcome, indicating that prejudices have been reduced to a larger extent. A formal power-analysis was not conducted because our selection criteria described above restricted the overall sample size.

Criteria for including a study in this analysis were similar to those of Study 1. To be included in the analysis an article had to state clearly in the title that the study is mainly about intergroup contact and prejudices or attitudes and to not refer to other major findings in the abstract, which could have been an alternative reason to cite the study. For example, an article with the title “Social contact as a variable in the expressed attitudes of normal adolescents toward EMR [educable mentally retarded] pupils” (Strauch, 1970) was included because it fulfilled the two criteria. However, an article with the title “Attitudes toward the mentally and physically disabled” (Furnham and Pendred, 1983) was not, because contact was not mentioned. Both abstracts of the just cited papers emphasized the points made. In total, 161 articles (31.26%) were included in the analysis, of which 22 were freely available (as in March 2017), 155 were single-study papers, and 100 have reported only one effect size relevant to the reduction of prejudice (i.e., 61 articles reported more than one; cf. Pettigrew and Tropp, 2006). We did not compare single-study papers with multi-study papers because of the highly unequal sample size (155 vs. 6; note that each multi-study paper had reported more than one relevant effect size).

#### Variables

Data for the same variables as in Study 1 were collected and all the analyses were again performed separately for CRs derived from GS and WoS. As in Study 1, all variables with the exception of free access were RIN transformed to normalize them. Additionally, the type of sample – children, adolescents, college students, and adults – was included as a predictor. We also tested whether the association between effect size and CR is stronger among those studies which reported an effect size compared to those who did not. For this, we relied on the meta-analysis of Pettigrew and Tropp (2006), who have reported which test has been used in the original paper. Finally, we tested whether the association between effect size and CR is stronger among those studies which reported only one effect size relevant for the contact hypothesis (vs. two or more), using the larger effect size (absolute value). If more than one key effect size is reported, it might be less clear why the paper was cited.

### Results

In a first step, differences on CR between the four sample types were investigated. Because none of the four univariate ANOVAs with the (transformed) CR from GS and WoS reached statistical significance (all *F*s < 0.39, *p*s > 0.76) the four conditions were collapsed and analyzed together.

#### Multiple Regressions

Regression analyzes with the transformed variables showed that the eight variables included explained 32 percent of the variance, with sample and effect size, and impact factor and year of publication being the only statistically significant predictors (see **Table 3**). Recall that the effect sizes have been coded in a way that smaller effect sizes (those toward -1) are more beneficial, hence effect sizes were expected to be a negative predictor of CR. However, if the transformed WoS CRs were used as dependent variable, effect size and year of publication were no longer significant predictors (β = -0.13, *p* = 0.16 and β = 0.16, *p* = 0.06), while sample size and impact factor remined significant (β = 0.25, *p* = 0.004 and β = 0.46, *p* < 0.001, respectively). The effects were mainly stronger in the predicted direction when we only included the 100 studies reporting one effect size relevant to the contact hypothesis: Effect and sample size did predict CRs as provided by GS (β = -0.31, *p* = 0.003 and β = 0.47, *p* < 0.001, respectively) and WoS (β = -0.28, *p* = 0.02 and β = 0.42, *p* < 0.001). In a final step, we excluded the four studies reporting effect sizes < -0.70, as effect sizes of this magnitude can indicate poor quality, because they are very unlikely to occur, given the distribution of effect sizes in psychology (Richard et al., 2003; Gignac and Szodorai, 2016). However, the pattern of results remained unchanged.

#### Correlational Analyses

Zero-order Pearson’s correlations revealed a somewhat different pattern (**Table 4**): sample size was correlated with CR as provided by both GS and WoS (*r*[159] = 0.22, *p* = 0.005 and *r*[132] = 0.20, *p* = 0.021, respectively), JIF, and estimated *p*-values (one tailed). However, effect sizes were uncorrelated to CR as provided by GS and WoS (*r*[159] = -0.08, *p* = 0.32 and *r*[132] = -0.08, *p* = 0.35, respectively; **Table 4**). The same pattern of results was found when Spearman’s rank correlation coefficients were computed with the untransformed variables.

**Table 4:** Pearson correlations of RIN transformed variables (Study 2).

		*M*	*SD*	1	2	3	4	5	6	7	8	9
1	Citation rate GS	86.01	160.73	1.00								
2	Citation rate WoS	37.49	70.15	0.96	1.00							
3	Effect size r	-0.25	0.20	-0.14	-0.11	1.00						
4	*p*-value	0.07	0.12	-0.31	-0.35	0.55	1.00					
5	Sample size	197.14	302.00	0.25	0.23	0.24	-0.12	1.00				
6	Free access	0.14	0.35	0.17	0.12	-0.09	-0.03	0.02	1.00			
7	Journal impact factor	1.56	1.09	0.41	0.44	0.04	-0.23	0.09	-0.06	1.00		
8	Number of authors	2.00	1.00	0.18	0.13	0.07	0.09	-0.03	0.15	0.05	1.00	
9	Position of 1st letter	11.19	7.03	-0.03	-0.05	0.00	0.04	0.06	0.02	0.17	-0.12	1.00
10	Year of publication	1983.88	11.90	0.24	0.15	-0.07	-0.02	0.03	0.21	-0.13	0.19	-0.08


*All variables are RIN transformed except ‘Free access’ before correlations were computed. Every |*r*|≥ 0.16 is significant on *p* < 0.05, |*r*|≥ 0.20 on *p* < 0.01 and every |*r*|≥ 0.26 on *p* < 0.001, two-tailed. *N* = 161 (for JIF: *N* = 123). GS, Google Scholar; WoS, Web of Science; Free access, 1: Articles is freely available, 0: is not; Position of 1st letter: Position of the first letter of the surname of the first author in the alphabet. Note that smaller effect sizes (i.e., toward -1) are considered to be more beneficial. Hence, negative relations between effect size and CRs were expected.*

However, despite the overall effect size was negative, some studies reported a positive effect size. This might have affected the results. We therefore first used the absolute value of the effect sizes and, in a second step, excluded all studies with positive effect sizes from the analysis. However, effect sizes were uncorrelated with the two measures of CRs. The effects were mainly stronger in the predicted direction when we only included the 100 studies reporting one effect size relevant to the contact hypothesis: The correlations of effect and sample size were *r*(98) = -0.13, *p* = 0.21 and *r*(98) = 0.63, *p* < 0.001 for GS CR and *r*(79) = -0.12, *p* = 0.28 and *r*(79) = 0.64, *p* < 0.001 for WoS CR. In a final step, we excluded the four studies reporting effect sizes < -0.70, as effect sizes of this magnitude can indicate poor quality. However, the pattern of results remained unchanged.

#### Interaction of Effect and Sample Size

To test for a potential interaction of effect and sample size, an interaction term was included. This was only done with the raw data, as transformations (both rank-based inverse normal and standardization) changes the sign of some values. For example, the mean of the raw effect sizes is -0.25, the mean of the transformed effect sizes by definition 0. The interaction term was significant for both CR from GS and WoS (β = -0.45, *p* = 0.017 and β = -0.48, *p* = 0.03, respectively), indicating that the relation between sample size and CR was moderated by effect size. Both, effect and sample size were no longer significant predictors on their own. For numerical smaller effect sizes (i.e., mostly statistically significant negative ones) the relation between sample size and CR was larger than for larger effect sizes. The effects were mainly stronger in the predicted direction when we only included the 100 studies reporting one effect size relevant to the contact hypothesis: The interaction term was again significant for both CR from GS and WoS (β = -0.68, *p* < 0.001 and β = -0.70, *p* < 0.001, respectively).

However, it is possible that the association between effect and sample size with CR emerges stronger for the 54 studies which have reported an effect size (correlation, proportion, or frequencies). However, this was not the case. Effect and sample size did not predict CRs as provided by GS (β = -0.31, *p* = 0.003 and β = 0.47, *p* < 0.001), and WoS (β = -0.28, *p* = 0.12 and β = 0.26, *p* = 15). Further, the correlations between effect and sample size remind non-significant for both CRs as provided by GS (*r*[52] = -0.05, *p* = 0.72 and *r*[52] = 0.09, *p* = 0.52) and WoS (*r*[45] = -0.07, *p* = 0.63 and *r*[45] = 0.08, *p* = 0.62). When we further excluded the studies which reported more than one focal effect size (leaving 31 studies), the correlations of effect size and CRs reminded non-significant for both GS and WoS CRs (*r*[29] = -0.05, *p* = 0.81 and *r*[23] = -0.09, *p* = 0.68), but sample size was again strongly correlated with CRs, both as provided by GS (*r*[29] = 0.62, *p* < 0.001) and WoS (*r*[23] = 0.67, *p* < 0.001).

#### Significant vs. Non-significant Results

Most studies reported statistically significant results (73.29%). *P*-values were estimated based on a directed hypothesis, i.e., we assumed that the null-hypothesis significance testing was done based on a one-tailed test. Significant results were cited significantly more often compared to non-significant (*t*[115.03] = 7.03, *p* < 0.001, *d* = 1.03). The pattern of results did not change if we assumed two-tailed testing (i.e., estimated two-tailed *p*-values), used CR according to WoS, or only focused on studies with one focal effect size.

#### Exploratory Analyses

Articles which were freely available in the internet were cited more often (*M* = 134.00, *SD* = 227.25) than those that were not (*M* = 78.42, *SD* = 147.25; *t*[29.50] = 2.25, *p* = 0.032, *d* = 0.48; note that we used the transformed CRs to perform our analyses). However, when the WoS CRs were used, this difference was no longer significant (*t*[26.10] = 1.39, *p* = 0.18). The pattern of results did not change when articles with more than one focal effect size were excluded, but were no longer significant for GS CRs as dependent variable when only those articles were analyzed, which reported an effect size (*t*[7.48] = 1.43, *p* = 0.19). However, the results must be interpreted carefully, because the sample of freely available articles was much smaller (22 vs. 139).

## General Discussion

We investigated the influence of effect and sample size on CR across two studies, both with and without controlling for several other potential predictors. The findings were mixed. Effect sizes predicted CR only in Study 2 and only while controlling for other variables. Sample size predicted in Study 1 and 2 CRs, when we controlled for other variables, but was only in Study 2 statistically significant correlated with CRs. The number of studies within the original papers, the number of tests regarding the focal test (ERS or contact hypothesis) within the original papers, and whether an effect size in the original study was reported did not affect the pattern of results. Further, the sample type did not have an influence on CR (only tested in Study 2).

The finding that effect sizes did not predict CR in Study 1 and only when controlling for other variables in Study 2, is surprising and needs further explanation. One possible explanation is that the majority of included articles across both studies did not report any effect size, instead reporting mean differences, among other statistics, making comparisons across several studies harder. Therefore, it remains unclear whether researchers do not value and/or compare effect sizes when deciding which methods to use, or whether researchers struggle in comparing effects when they presented with different effect sizes or without any effect size at all. In contrast, sample sizes are easy to see and compare. However, these are only *post hoc* speculations based on descriptive results. For example, in Study 2, the correlation coefficient between effect size and CR (0.14, recoded) did not differ from the correlation between sample size and CR (0.25), as a comparison of dependent correlation coefficients revealed (*Z* = 0.91, *p* = 0.36).

Our findings with regard to the effect size in psychology are mainly congruent with the literature: studies from ecology have also only found inconclusive weak positive correlations with CR (Leimu and Koricheva, 2005; Barto and Rillig, 2012). The neglect of effect and sample size while deciding what to cite is in line with numerous findings showing that psychological studies are underpowered, i.e., the sample size is too small for the effect they wish to detect (Sedlmeier and Gigerenzer, 1989; Fraley and Vazire, 2014). If statistical power would be valued more, studies with larger sample sizes would likely been more acknowledged.

The sample size predicted the CR in studies exploring the contact hypothesis stronger than in studies exploring emotion regulation strategies. Although the difference between the correlation coefficients of Study 1 and 2 regarding the sample size-CR association was not statistically significant (*r* = 0.08 vs. 0.25, *Z* = -1.50, *p* = 0.13), they are in line with the literature. This (descriptive) difference could be due to different research designs: studies exploring emotion regulation strategies are usually laboratory experiments, whereas studies exploring the contact hypothesis are often field studies or field experiments. That is, studies in natural settings. Studies that investigated the predictors of CRs in medical journals, where research is predominantly conducted in natural settings, also reported a positive influence of sample size on predictions (Callaham et al., 2002; Kostoff, 2007; Farshad et al., 2013) (but see Etter and Stapleton, 2009, for an exception), unlike studies in the field of ecology or management (Leimu and Koricheva, 2005; Bergh et al., 2006; Barto and Rillig, 2012). This can indicate that in some scientific (sub-)fields and topical areas larger sample sizes and well-powered studies are considered as more important. However, this is of course *post hoc* speculation, based on descriptive results. There are many more differences between Study 1 and 2 than the setting of the studies, including larger variances in the sample sizes or the year of publication (most papers used in Study 1 were published post-2000, most papers used in Study 2 pre-2000; cf. **Tables 2**, **4**).

In line with previous findings (Lawrence, 2001; Piwowar et al., 2007; Vanclay, 2013) we found that freely available articles were cited more often (at least for CRs as provided by GS in Study 2). However, as Gaulé and Maystre (2011) have argued, this may very well be due to differences in quality: articles with higher quality as judged by experts within a given field, were cited more often and more likely published open access in one specific selected journal (*PNAS*). However, explorative *post hoc* results of our two studies show that the importance of published articles is independent of open access publications. Effect sizes were larger for the freely available studies, but only in Study 1 (*t*[98.26] = 2.19, *p* = 0.031, *d* = -0.39) and not Study 2 (*t*[28.23] = 1.15, *p* = 0.26, *d* = 0.26). Sample sizes did not differ between freely and non-freely available articles across both studies (*p*s > 0.70). The only statistical significant difference was no longer statistically significant, after controlling for multiple comparisons, for most of the common controlling procedures such as Bonferroni or Sidak corrections.

### Limitations

A potential objection against our rationale is that articles about phenomena studied for the very first time are cited more often because they are providing a new method, even if effect and sample size are smaller. Hence, our results could be biased, because we did not control for whether a paper provides a new method. However, we doubt that such studies have systematic smaller effect and sample sizes. If anything, the effect sizes are larger. It has been argued that effect sizes decline on average in subsequent studies (“decline-effect”; Schooler, 2011).

Another limitation pertains the sample size of both studies. The sample size might have been too small to detect a potential small effect. In both studies, the correlation between effect size and CR was in the expected direction, which might indicate the existence of a very small effect. We had intentionally selected meta-analyses containing large samples and drawn all studies which fulfilled our inclusion criteria. Although we believe that we have justified this approach in the Introduction, it restricts the sample size. However, it is not clear whether there is a topical area in which larger effect sizes are considered to be more beneficial and which contains enough studies so a well-powered replication of our findings would be possible. Also, it is unclear whether such a small effect, once confirmed, would indeed be meaningful. Instead, we believe that an effect around the one found in Study 1 and 2 (*r* ≈0.10) would be negligible, because, based on our reasoning presented in the Introduction, the effect should be at least medium.

A further limitation relates to the reproducibility of the variable free availability in both studies. Recall that we chose to use GS to check whether an article is freely available. However, researchers might have added their articles (or pre-/post-prints) add any point since we have done the data collection in spring 2017, or the former freely available articles might have been removed (e.g., because of copyright infringements). Although our approach is consistent with the literature (e.g., Lawrence, 2001; Hajjem et al., 2006), the raise of Sci-Hub, a website which hosts millions of pirated papers and grants free access to them, might bias previous findings related to open access. Alone between October 2015 and March 2016 28 million articles were downloaded from Sci-Hub (Bohannon, 2016). In other words, whether an article is freely available on GS or anywhere else in the web becomes less important because most published articles are freely available through Sci-Hub.

## Conclusion

The partly mixed findings of Study 1 and Study 2 demonstrate that the amount of explained variance of variables which predict CRs do not only vary between numerous scientific fields, but also between topical areas within one scientific field. Variables, which are rather unrelated to the quality or importance of studies can predict CRs (e.g., journal impact factor; cf. Brembs et al., 2013), as do variables which are related to the quality and importance of studies (see Introduction). Given the inconclusive findings of Study 1 and 2 combined, they challenge the widespread use of CRs as a measure of quality because it remains unclear how much bias and error CRs contain. We therefore think a more open and widespread discussion of how to estimate quality, novelty, and importance of research can yield to less biased measures that can complement CRs (e.g., Bornmann and Marx, 2014).

## Author Contributions

Conceived: PH. Designed the Studies: PH and JH. Collected and analyzed the data: PH and JH. Wrote the paper: PH and JH.

## Conflict of Interest Statement

The authors declare that the research was conducted in the absence of any commercial or financial relationships that could be construed as a potential conflict of interest.

## Footnotes

1 http://scholar.google.com/

2 https://osf.io/mra24/

3 https://www.webofknowledge.com/

## References

[B1] American Psychology Association [APA] (2010). *Publication Manual of the American Psychology Association*, 6th Edn Washington, DC: American Psychology Association.

[B2] Archambault É. Campbell D. Gingras Y. Larivière V. (2009). Comparing bibliometric statistics obtained from the web of science and scopus. *J. Am. Soc. Inform. Sci. Technol.* 60 1320–1326. 10.1002/asi.21062

[B3] Aulchenko Y. S. Ripke S. Isaacs A. van Duijn C. M. (2007). GenABEL: an R library for genome-wide association analysis. *Bioinformatics* 23 1294–1296. 10.1093/bioinformatics/btm108 17384015

[B4] Ayres I. Vars F. E. (2000). Determinants of citations to articles in elite law reviews. *J. Legal Stud.* 29 427–450. 10.1086/468081

[B5] Baldi S. (1998). Normative versus social constructivist processes in the allocation of citations: a network-analytic model. *Am. Sociol. Rev.* 63 829–846.

[B6] Barto E. K. Rillig M. C. (2012). Dissemination biases in ecology: effect sizes matter more than quality. *Oikos* 121 228–235. 10.1111/j.1600-0706.2011.19401.x

[B7] Bergh D. D. Perry J. Hanke R. (2006). Some predictors of SMJ article impact. *Strateg. Manag. J.* 27 81–100. 10.1002/smj.504

[B8] Bishara A. J. Hittner J. B. (2012). Testing the significance of a correlation with nonnormal data: comparison of pearson, spearman, transformation, and resampling approaches. *Psychol. Methods* 17 399–417. 10.1037/a0028087 22563845

[B9] Bohannon J. (2016). Who’s downloading pirated papers? Everyone. *Science* 352 508–512. 10.1126/science.352.6285.508 27126020

[B10] Bornmann L. Marx W. (2014). The wisdom of citing scientists. *J. Assoc. Inform. Sci. Technol.* 65 1288–1292. 10.1002/asi.23100

[B11] Bornmann L. Mutz R. Daniel H.-D. (2010). A reliability-generalization study of journal peer reviews: a multilevel meta-analysis of inter-rater reliability and its determinants. *PLoS ONE* 5:e14331 10.1371/journal.pone.0014331 PMC3001856 21179459

[B12] Brembs B. Button K. Munafò M. (2013). Deep impact: unintended consequences of journal rank. *Front. Hum. Neurosci.* 7:291 10.3389/fnhum.2013.00291 PMC3690355 23805088

[B13] Button K. S. Ioannidis J. P. A. Mokrysz C. Nosek B. A. Flint J. Robinson E. S. J. (2013). Power failure: why small sample size undermines the reliability of neuroscience. *Nat. Rev. Neurosci.* 14 365–376. 10.1038/nrn3475 23571845

[B14] Callaham M. Wears R. L. Weber E. (2002). Journal prestige, publication bias, and other characteristics associated with citation of published studies in peer-reviewed journals. *JAMA* 287 2847–2850. 10.1001/jama.287.21.2847 12038930

[B15] Cohen J. (1992). A power primer. *Psychol. Bull.* 112 155–159. 10.1037/0033-2909.112.1.155 19565683

[B16] Didegah F. Thelwall M. (2013a). Determinants of research citation impact in nanoscience and nanotechnology. *J. Am. Soc. Inform. Sci. Technol.* 64 1055–1064.

[B17] Didegah F. Thelwall M. (2013b). Which factors help authors produce the highest impact research? Collaboration, journal and document properties. *J. Informetr.* 7 861–873. 10.1016/j.joi.2013.08.006

[B18] Etter J.-F. Stapleton J. (2009). Citations to trials of nicotine replacement therapy were biased toward positive results and high-impact-factor journals. *J. Clin. Epidemiol.* 62 831–837. 10.1016/j.jclinepi.2008.09.015 19128941

[B19] Eyre-Walker A. Stoletzki N. (2013). The assessment of science: the relative merits of post-publication review, the impact factor, and the number of citations. *PLoS Biol.* 11:e1001675 10.1371/journal.pbio.1001675 PMC3792863 24115908

[B20] Fanelli D. (2010). Positive” results increase down the hierarchy of the sciences. *PLoS ONE* 5:e10068 10.1371/journal.pone.0010068 PMC2850928 20383332

[B21] Fanelli D. (2013). Positive results receive more citations, but only in some disciplines. *Scientometrics* 94 701–709. 10.1007/s11192-012-0757-y

[B22] Farshad M. Sidler C. Gerber C. (2013). Association of scientific and nonscientific factors to citation rates of articles of renowned orthopedic journals. *Eur. Orthop. Traumatol.* 4 125–130. 10.1007/s12570-013-0174-6

[B23] Fraley R. C. Vazire S. (2014). The N-pact factor: evaluating the quality of empirical journals with respect to sample size and statistical power. *PLoS ONE* 9:e109019 10.1371/journal.pone.0109019 PMC4189949 25296159

[B24] Furnham A. Pendred J. (1983). Attitudes towards the mentally and physically disabled. *Br. J. Med. Psychol.* 56 179–187. 10.1111/j.2044-8341.1983.tb01545.x 6224507

[B25] Gaulé P. Maystre N. (2011). Getting cited: does open access help? *Res. Policy* 40 1332–1338. 10.1016/j.respol.2011.05.025

[B26] Gignac G. E. Szodorai E. T. (2016). Effect size guidelines for individual differences researchers. *Pers. Individ. Dif.* 102 74–78. 10.1016/j.paid.2016.06.069

[B27] Gottfredson S. D. (1978). Evaluating psychological research reports: dimensions, reliability, and correlates of quality judgments. *Am. Psychol.* 33 920–934. 10.1037/0003-066X.33.10.920

[B28] Hajjem C. Harnad S. Gingras Y. (2006). *Ten-Year Cross-Disciplinary Comparison of the Growth of Open Access and How it Increases Research Citation Impact.* Available at: http://arxiv.org/abs/cs/0606079

[B29] Hanssen T.-E. S. Jørgensen F. (2014). Citation counts in transportation research. *Eur. Trans. Res. Rev.* 6 205–212. 10.1212/WNL.0b013e31821d74fa

[B30] Haslam N. Ban L. Kaufmann L. Loughnan S. Peters K. Whelan J. (2008). What makes an article influential? Predicting impact in social and personality psychology. *Scientometrics* 76 169–185. 10.1007/s11192-007-1892-8

[B31] Hegarty P. Walton Z. (2012). The consequences of predicting scientific impact in psychology using journal impact factors. *Pers. Psychol. Sci.* 7 72–78. 10.1177/1745691611429356 26168426

[B32] Judge T. A. Cable D. M. Colbert A. E. Rynes S. L. (2007). What causes a management article to be cited: article, author, or journal? *Acad. Manag. J.* 50 491–506. 10.2307/20159868

[B33] Kostoff R. N. (2007). The difference between highly and poorly cited medical articles in the journal *Lancet*. *Scientometrics* 72 513–520. 10.1007/s11192-007-1573-7

[B34] Kulkarni A. V. Busse J. W. Shams I. (2007). Characteristics associated with citation rate of the medical literature. *PLoS ONE* 2:e403 10.1371/journal.pone.0000403 PMC1852582 17476325

[B35] Kunzmann U. Kupperbusch C. S. Levenson R. W. (2005). Behavioral inhibition and amplification during emotional arousal: a comparison of two age groups. *Psychol. Aging* 20 144–158. 10.1037/0882-7974.20.1.144 15769220

[B36] Laband D. N. Piette M. J. (1994). The relative impacts of economics journals: 1970-1990. *J. Econ. Literat.* 32 640–666.

[B37] Lawrence S. (2001). Free online availability substantially increases a paper’s impact. *Nature* 411 521. 10.1038/35079151 11385534

[B38] Leimu R. Koricheva J. (2005). What determines the citation frequency of ecological papers? *Trends Ecol. Evolut.* 20 28–32. 10.1016/j.tree.2004.10.010 16701337

[B39] Masicampo E. J. Lalande D. R. (2012). A peculiar prevalence of p values just below.05. *Q. J. Exp. Psychol.* 65 2271–2279. 10.1080/17470218.2012.711335 22853650

[B40] Meho L. I. Yang K. (2007). Impact of data sources on citation counts and rankings of LIS faculty: web of science versus scopus and google scholar. *J. Am. Soc. Inform. Sci. Technol.* 58 2105–2125. 10.1002/asi.20677

[B41] Mingers J. Xu F. (2010). The drivers of citations in management science journals. *Eur. J. Oper. Res.* 205 422–430. 10.1016/j.ejor.2009.12.008

[B42] Newman J. M. Cooper E. (1993). Determinants of academic recognition: the case of the journal of applied psychology. *J. Appl. Psychol.* 78 518–526. 10.1037/0021-9010.78.3.518

[B43] Niu X. Hemminger B. M. (2012). A study of factors that affect the information-seeking behavior of academic scientists. *J. Am. Soc. Inform. Sci. Technol.* 63 336–353. 10.1002/asi.21669

[B44] Nosek B. A. Spies J. R. Motyl M. (2012). Scientific Utopia II. Restructuring incentives and practices to promote truth over publishability. *Perspect. Psychol. Sci.* 7 615–631. 10.1177/1745691612459058 26168121 PMC10540222

[B45] Open Science Collaboration (2015). Estimating the reproducibility of psychological. *Science* 349:aac4716 10.1126/science.aac4716 26315443

[B46] Peters H. P. F. van Raan A. F. (1994). On determinants of citation scores: a case study in chemical engineering. *J. Am. Soc. Inform. Sci.* 45 39–49.

[B47] Pettigrew T. F. Tropp L. R. (2006). A meta-analytic test of intergroup contact theory. *J. Pers. Soc. Psychol.* 90 751–783. 10.1037/0022-3514.90.5.751 16737372

[B48] Petty R. E. Cacioppo J. T. (1986). “The elaboration likelihood model of persuasion,” in *Advances in Experimental Social Psychology* Vol. 19 ed. Berkowitz L. (New York, NY: Academic Press), 123–205.

[B49] Piwowar H. A. Day R. S. Fridsma D. B. (2007). Sharing detailed research data is associated with increased citation rate. *PLoS ONE* 2:e308 10.1371/journal.pone.0000308 PMC1817752 17375194

[B50] Richard F. D. Bond C. F. Jr. Stokes-Zoota J. J. (2003). One hundred years of social psychology quantitatively described. *Rev. Gen. Psychol.* 7 331–363. 10.1037/1089-2680.7.4.331

[B51] Salsburg D. S. (1985). The religion of statistics as practiced in medical journals. *Am. Stat.* 39 220–223. 10.1080/00031305.1985.10479435

[B52] Schooler J. (2011). Unpublished results hide the decline effect. *Nat. News* 470 437–437. 10.1038/470437a 21350443

[B53] Sedlmeier P. Gigerenzer G. (1989). Do studies of statistical power have an effect on the power of studies? *Psychol. Bull.* 105 309–316. 10.1037/0033-2909.105.2.309

[B54] Sharpe D. (2013). Why the resistance to statistical innovations? Bridging the communication gap. *Psychol. Methods* 18 572–582. 10.1037/a0034177 24079924

[B55] Strauch J. D. (1970). Social contact as a variable in the expressed attitudes of normal adolescents toward EMR pupils. *Except. Child.* 36 495–500. 5451991

[B56] Stremersch S. Verniers I. Verhoef P. C. (2007). The quest for citations: drivers of article impact. *J. Mark.* 71 171–193.

[B57] Tregenza T. (1997). Darwin a better name than Wallace? *Nature* 385 480 10.1038/385480a0 9020349

[B58] Uthman O. A. Okwundu C. I. Wiysonge C. S. Young T. Clarke A. (2013). Citation classics in systematic reviews and meta-analyses: who wrote the top 100 most cited articles? *PLoS ONE* 8:e78517 10.1371/journal.pone.0078517 PMC3796507 24155987

[B59] Vanclay J. K. (2013). Factors affecting citation rates in environmental science. *J. Informetr.* 7 265–271. 10.1016/j.joi.2012.11.009

[B60] Wang J. Shapira P. (2015). Is there a relationship between research sponsorship and publication impact? An analysis of funding acknowledgments in nanotechnology papers. *PLoS ONE* 10:e0117727 10.1371/journal.pone.0117727 PMC4335067 25695739

[B61] Webb T. L. Miles E. Sheeran P. (2012). Dealing with feeling: a meta-analysis of the effectiveness of strategies derived from the process model of emotion regulation. *Psychol. Bull.* 138 775–808. 10.1037/a0027600 22582737

[B62] Wong Q. J. J. Moulds M. L. (2009). Impact of rumination versus distraction on anxiety and maladaptive self-beliefs in socially anxious individuals. *Behav. Res. Ther.* 47 861–867. 10.1016/j.brat.2009.06.014 19608157

